# Role of Sn as a Process Control Agent on Mechanical Alloying Behavior of Nanocrystalline Titanium Based Powders

**DOI:** 10.3390/ma13092110

**Published:** 2020-05-02

**Authors:** Izabela Matuła, Maciej Zubko, Grzegorz Dercz

**Affiliations:** 1 Institute of Materials Engineering, University of Silesia in Katowice, 75 Pułku Piechoty 1a, 41-500 Chorzów, Poland; maciej.zubko@us.edu.pl; 2 Department of Physics, Faculty of Science, University of Hradec Králové, Rokitanského 62, 500 03 Hradec Králové, Czech Republic

**Keywords:** Sn, process control agent, nanocrystalline Ti based alloy, mechanical alloying, Rietveld method

## Abstract

In this study, the effects of Sn as a process control agent (PCA) on the final powder sizes, morphology, homogenization and alloying process of a new titanium alloy were investigated. Two kinds of powders, Ti10Ta8Mo and Ti10Ta8Mo3Sn (wt %), were prepared using a mechanical alloying process. For the Ti10Ta8Mo3Sn (wt %) alloy, the Sn element was used as PCA to enhance the milling process in the planetary ball mill. The milling process of both compositions was carried out with 200 rpm for 10, 15, 20, 40, 60, 80 and 100 h. The results confirmed that using Sn as a process control agent can result in a relatively good size distribution and better yield performance compared to samples without Sn addition. The phase analysis using X-ray diffraction proved the formation of the α nanocrystalline phase and the partial phase transformation from α to nanocrystalline β phases of both alloy compositions. The Scaning Electron Micoscope- Backscattered Electrons SEM-BSE results confirmed that the use of Sn as the PCA can provide a better homogenization of samples prepared by at least 60 h of ball milling. Furthermore, the presence of Sn yielded the most uniform, spheroidal and finest particles after the longest milling time.

## 1. Introduction

Considered to be an unconventional production method, the ball mill is a simple and cost-effective way of producing homogeneous and ultrafine powders in small production runs [1,2]. Mechanical Alloying (MA) is a method of processing a powder without liquefaction, which consists in the repetitive cold welding and cracking of particles as a result of the reciprocal collision of milling balls and powder particles [1,3,4,5]. Cold welding of different powder particles takes place when particles mutually penetrate each other after subsequent collisions with the balls [6]. The fracture process occurs when the larger particles disintegrate into smaller pieces due to overloading as a result of continuous collisions [7,8]. Depending on the ductility of the used material being milled, fracturing or cold welding can dominate and result in the formation of a lot of small particles, or when there is complete cold welding with the balls and the walls of the milling bowls the process′s efficiency and material recovery are negatively affected [3,9,10,11,12]. Therefore, maintaining a suitable relationship regarding the cold welding and fracturing phenomena ensures a stable particle size. Furthermore, the maintenance of said balance allows the milling balls to mill the powder continuously without slowing or halting the process. Therefore, in order to ensure that the milling process proceeds correctly, it is necessary to use additional lubricants as process control agents (PCAs) [10]. The use of a PCA usually leads to homogenous and fine particles as well as a reduction in the crystallite size of the powders to a nanometer scale. This is due to the fact that, in the case of a planetary ball mill, the mechanical alloying of the material results mainly from the strong interactions of the material with the balls during the rotation of the bowls [13,14]. In addition, MA is a process that allows materials of different structure types to result in amorphous [15], nanocrystalline [12,16,17] and porous materials [13,17,18,19].

The PCA is added to the starting powders for the milling process to limit the effect of cold welding. This is due to the fact that true alloying of powders can occur only when the right balance between cold welding and fracturing of powder particles is maintained [20].

Where ductile metals are milled, the PCA should be used to reduce excessive cold welding, which can lead to excessive particles. PCAs are typically of organic origin and adsorb on the surface of the metal, reducing the surface stress of the milled powders by limiting the pure metal–metal contact [21,22,23]. The type of PCA also affects the balance of cold welding and cracking of particles and may be stearic acid [24], methyl alcohol [25], ethyl alcohol [26], polyethylene glycol [27], calcium [28] or magnesium. In MA, the PCA is usually used in an amount from 1 to 5 wt %. total mass of the powder. We use olefinic PCAs, because we limit the possibility of introducing elements such as O, N, H and C into the mixture, occupying interstitial positions, which is potentially unfavorable to the final material as a result of the formation of oxides, nitrides, hydrides and carbides during the sintering process. Due to the challenging local conditions (mechanical and thermal) at the moment when the balls collide, milled powders are decomposed, and additionally they become contaminated as result of interactions between PCAs and the milled material [7,29,30,31]. In previous studies, it was noted that Mg [32] or Ca [7] and all rare earth(RE) metals [33] have low dissolution rates in titanium, thus delaying the phenomenon of cold welding. Therefore, it is extremely hard to find optimal PCAs that would allow a high efficiency of mechanical titanium alloying while maintaining a similar chemistry. Based on the above, the authors used Sn as the PCA for the mechanical alloying of titanium. In recent years, Sn has been classified as a neutral alloying element for titanium alloys [34,35,36]. It has been reported in the literature that Sn has no noticeable impact on the stability of the α or β phases. Sn forms solid solutions with Ti, and hence it is considered a neutral element [37,38]. In addition, it was noted that the presence of Sn limits the creation of an unfavorable ω-phase in titanium alloys [39,40]. High-dissolution of Sn in Ti (up to about 20% by weight) provides relatively high possibilities in terms of the amount of PCA used [41]. However, in order to reduce the undesirable effects, it is assumed that the content is limited to 5% by weight [42]. Additionally, different authors [38] showed that Sn (<5 wt %) in the cast Ti–Sn binary alloy clearly improved Young’s modulus and bending strength. In summary, the addition of 3 wt % Sn proved to be a very good way to maximize the powder yield and stabilize the alloy microstructure [41,43,44,45,46]. While PM studies on the addition of Sn in Ti are relatively limited, generally demonstrating minimal benefits, Sn additives for biomedical applications were studied [47,48]. The use of Ti alloy with Sn, Mo and Ta alloying elements in orthopedic biomaterials has been previously investigated, and the biocompatibility, mechanical properties and corrosion resistance of Ti alloys [15,32,49,50] have been reported as being improved.

The aim of this study was to investigate the influence of pure Sn as a PCA on the structural characteristics, morphology and alloying process of precursors of two kinds of powders, Ti10Ta8Mo and Ti10Ta8Mo3Sn, with Sn as the PCA. One PCA quantity was used to ensure the high efficiency of the milling process, uniform morphology of the powder and appropriate particle size, with the objective of producing a new type of porous titanium biomedical alloy. These studies are designed to yield nanocrystalline and homogeneous pre-alloyed powders for use in further synthesis by sintering or additive techniques.

## 2. Experimental Details

### Specimen Preparation

The Ti10Ta8Mo and Ti10Ta8Mo3Sn (wt %) (samples labeled TTM and TTM3S, respectively) alloy compositions were obtained from commercial powders; Ti (Atlantic Equipment Engineers (AEE, New York, NY, USA), 99.7%, < 45 μm), Nb (AEE, 99.8%, < 5μm), Mo (AEE, New York, NY, USA, purity 99.7%, particle size ≤ 2 µm) and Sn (Sigma Aldrich, Darmstand, Germany, purity 99.9%, ≤ 5 µm). The nanocrystalline powders were formed in a high-energy milling mill (Fritsch Pulverisette 7 premium line) in a gas-protective atmosphere (Ar). The powders were fresh each time and were operated in a glove box under Ar protective gas atmosphere. Table 1 summarizes the parameters of the mechanical alloying process. The milling bowls and balls were made of hardened steel (AISI 52100). Table 2 shows the chemical composition of the AISI 52100 steel balls.

The qualitative phase analysis and structure of the powders after the milling process were conducted using X-ray diffraction by a Phillips X′Pert diffractometer made in Almelo, Holland (CuKα − λ = 1.54178 Ǻ) with the following operating parameter: 30 mA and 40 kV, steps of 0.04° (2*θ*) and an angle range of 20° to 140° (2θ). The LaB6 powder (SRM660a) was applied as a reference material for the linear profile to measure the instrumental broadening. The accuracies of the unit cell parameters were measured using an α-Al_2_O_3_ plate standard (SRM 1976) and found to be ±0.02%. The parameters of profiles of particular diffraction peaks were defined by the Toray PRO-FIT method [51], which uses the Pearson VII function to fit the lines. Rietveld analysis was carried out with the DBWS-9807 program, which is an updated version of DBWS programs for improving Rietveld with PC and mainframe [52]. The pseudo-Voigt function proved to be the most useful in describing the profiles of diffraction lines in the Rietveld analysis [53,54]. The quantitative analysis of the phase was conducted using the relationship proposed by Hill and Howard [55,56]. The crystallite sizes and lattice distortion of the α and β phases were determined by the Williamson−Hall method [57].

A microscopic analysis of crystallites was carried out using at transmission electron microscope JEM 3010, operating at an acceleration voltage of 300 kV. The morphology of the powders was analyzed by a scanning electron microscope JEOL JSM 6480 (Tokyo, Japan, accelerating voltage of 20 kV). A chemical analysis was carried out using an X-ray detector (EDS) manufactured by IXRF (Houston, TX, USA) using a traditional/standard calibration method.

## 3. Results and Discussion

**Figure 1:**
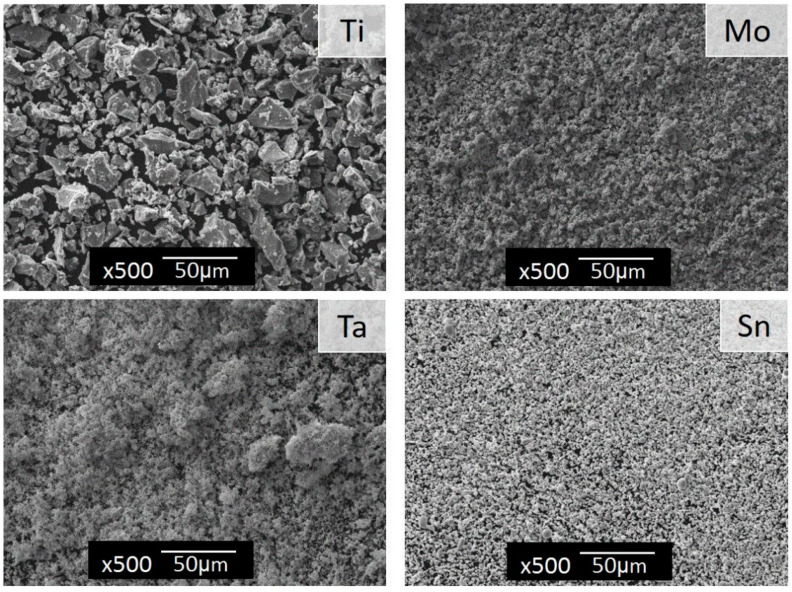
Scanning Electron Microscope (SEM) micrographs of the initial powders, Ti, Ta, Mo and Sn, showing different particle morphologies. The scale bar represents 50 μm.

The morphology of the commercial powders is presented in Figure 1. The powders showed a significant difference in their morphology and size. Titanium showed irregular shapes of sharp-edged particles and a broader size distribution. Most particles of Ti had a size below 50 µm. In contrast, tantalum, molybdenum and tin had very fine particles, and Ta and Mo were observed to have formed agglomerates.

**Figure 2:**
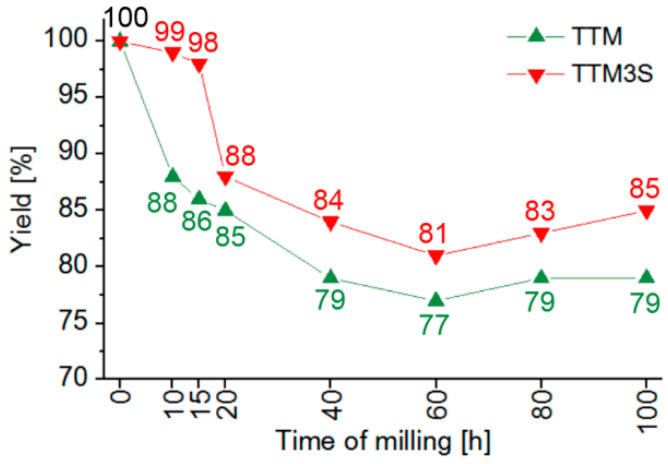
Process yield characteristics of Ti10Ta8Mo and Ti10Ta8Mo3Sn (wt %) powders after 10, 15, 20, 40, 60, 80 and 100 h of milling.

An important parameter of the milling process is the powder yield. Figure 2 shows the graph depicting the relationship between the yield and milling time of powders with and without Sn as a PCA. The milling results clearly show that tin is a highly efficient PCA for titanium milling.

The graph clearly shows that for powder without Sn as a PCA, the cold welding process dominated. The results show that the obtained yield mainly depends on the applied milling time. For the two shortest milling times (10 and 15 h), the yields of the non-PCA powder were 88% and 86%, respectively. On the other hand, when using 3 wt % tin as a PCA, there was a noticeable (above 10%) increase in the powder yield. Excellent yields of 99% and 98% were obtained for 10 and 15 h of milling (Figure 2), respectively.

In the case of the two longest milling times for both the Ti10Ta8Mo and Ti10Ta8Mo3Sn samples, there was a slight increase in the yield of powders due to the detachment of part of the welded coating from the balls and the walls of the milling bowls. This was probably due to the increase of stress as a result of the prolonged and continuous impacts of the milling balls.

Unfortunately, for a milling time between 15 and 20 h, the tin used as a PCA did not completely limit the cold welding, resulting in a decrease of the process yield. On the other hand, as shown in other studies, the use of magnesium as a PCA also has a beneficial effect in terms of a fast and cheap alloying powder formation process. Adamek [32] showed that the use of Mg (10%, 15%, 20%) results in about a 94%–96% yield after 15 h of milling. After 100 h of milling, powder yields of over 90% were obtained for all the studied materials. Notably, the yield was found to increase with an increasing Mg content. Unfortunately, the medical use of magnesium is limited because, at a physiological pH, magnesium alloys quickly corrode. This can lead to muscular paralysis, hypotension and respiratory failure, as well as cardiac arrest [58,59,60,61]. In contrast, the presence of tin is neutral for the human body.

**Figure 3:**
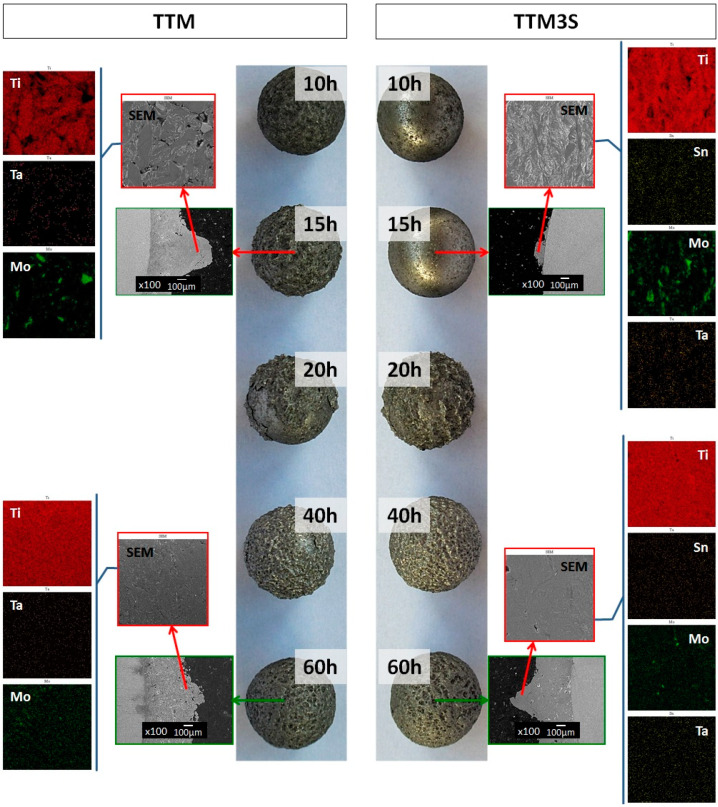
The balls and cross-sections of the balls with the milling product obtained during the milling process, with the distribution of Ti, Ta, Mo and Sn elements observed on the surface of the ball.

**Figure 4:**
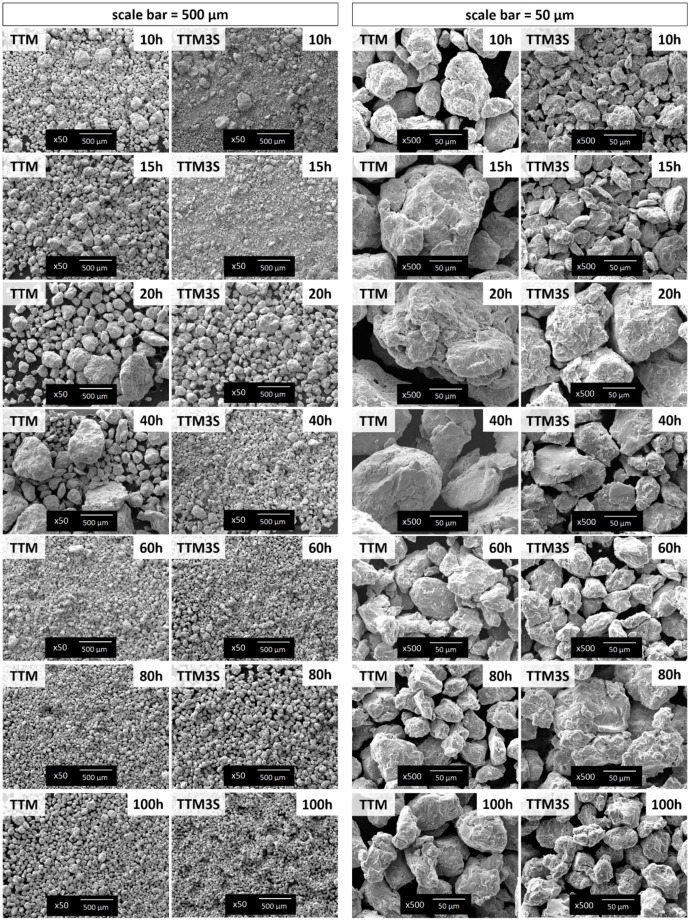
SEM images of TTM and TTM3S powders after 10, 15, 20, 40, 60, 80 and 100 h of milling: **left side**—scale bar represents 500 μm; **right** **side**—scale bar represents 50 μm.

However, the yield parameter is not the only indicator of a properly mechanically alloyed powder. Figure 3 shows the surface of balls with the material, which became welded onto the balls at each milling stage. It is clear that the welding and fracturing processes alternate (e.g., balls for 20 h of TTM powder). It should be highlighted that there was a lack of powder deposited on the balls’ surface after 10 and 15 h of milling for the TTM3S series. SEM images of the cross-section of the balls used for 15 h and 60 h milling with the cold-welded material (Figure 3) revealed further stages and processes which occur during the successive cold welding of the material onto the balls. The distribution map of the elements shows the cold welding of initial elements and their alloys in a layered manner. This indicates that an incomplete alloying process occurred. Similar observations of the cross-section of the balls were made by Dercz et al. [3,62]. In addition, the mentioned studies have shown that there is no diffusion of Fe and C into the milled material. Observations of the microstructure showed that, after 15 h of milling of the initial powders, the materials were partially synthesized. For the longer milling time, the distribution map of the elements shows a rather uniform microstructure, indicating that a superior alloying process occurred. It was found that prolonging the milling time caused a higher fractionation of the powder and reduction of the surface area of one element concentration. A comparison of the morphologies of TTM and TTM3S powders at different milling stages is presented in Figure 4.

The SEM images show consecutive changes in the morphology of powders with an increasing milling time. It should be noted that, for TTM powders during milling, significant changes in the morphology of powder particles were observed and consisted mainly of two stages. SEM images from the initial grinding stage show the gradual enlargement of particles, from initial powders with a different morphology to the synthesis of polygonal particles (from 10 to 20 h). At the second stage (above 40 h of milling), the particle size gradually decreased, and eventually a homogenous and finer powder was obtained. For the Ti10Ta8Mo3Sn powder, the formation of polygonal particles took place from 20 h onwards (Figure 4). For two (20 and 40 h) milling times of TTM powders, the formation of large particles with a size of up to 500 μm was visible. On the other hand, for TTM3S powders, further fracturing and comminuting of particles were observed. For longer milling times (>40 h) of TTM and TTM3S powders, the powders were brittle and the fracturing process was significant, as a result of which a change in the particle morphology is seen. The morphologies of both powder compositions after long milling time processes were similar, except that the particles sizes were smaller owing to the use of tin as a PCA. For the longest milling time, the milling process regulated the particle size of both components. It should be mentioned that finer TTM3S powder particles were obtained in contrast to TTM powder. The formation of fine, spheroidal particles and less variation in composition between particles for longer milling times resulted in better homogeneity. The increase in particle spheroidization was the result of the minimization of the Gibbs powder-free energy during the mechanical alloying process [63]. Some scientific works noted that the mechanical alloying process causes the powder particles to undergo multiple flattening, cold welding, fracturing and, again, welding processes. Cold welding or fracturing processes may be predominant at each stage and depend mainly on the deformations typical of the starting powders and their kinetics [1,63,64]. It has been found that the most uniform, spheroidal and finest powder particles were observed for the TTM3S powder obtained after the longest milling time. This is a good prognostic for further processing in powder metallurgy, because it will potentially enable a homogenous solid with a low porosity to be obtained.

**Figure 5:**
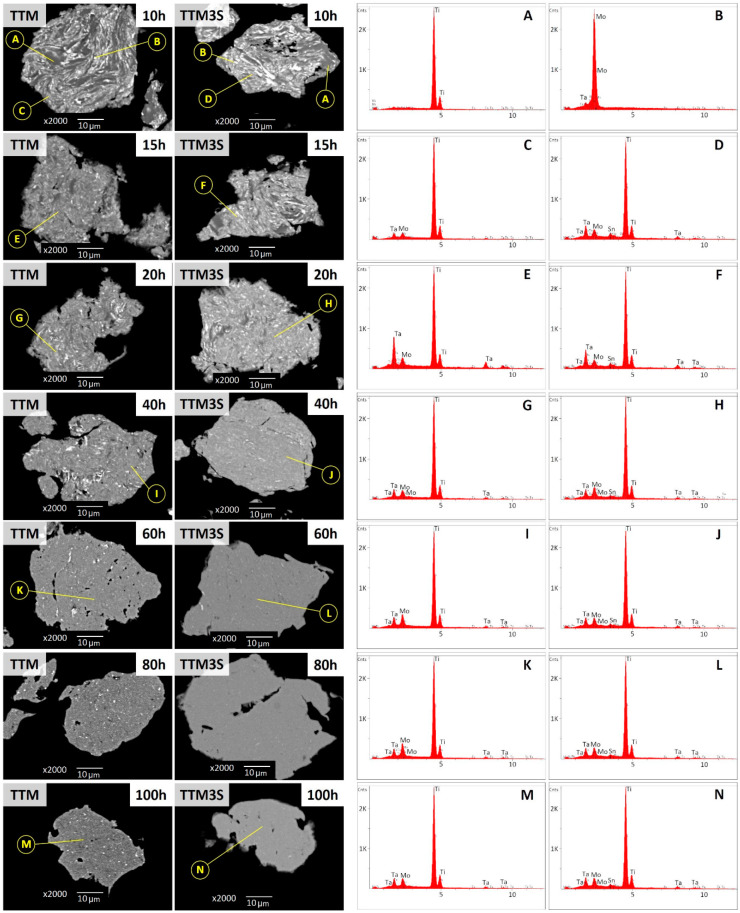
SEM-Backscattered electrons images and SEM-EDS point analysis of TTM and TTM3S powders after 10, 15, 20, 40, 60, 80 and 100 h of milling.

To better appreciate the influence of the tin on the microstructure, SEM-BEI backscattered electron images of both types of powders were performed for different ball milling times. The components can be distinguished by regions of light and dark contrast using SEM-BEI, while the elemental composition of these regions is determined by SEM-EDS analysis. As can be seen on the cross-section of the milled powders, the formation of the alloy was strongly dependent on the ball milling time and the presence of tin (Figure 5). Image analysis showed that after 10 and 15 h of milling of both types of powders, the Ti and Mo fragments were trapped inside the particles (Figure 5 (point A) and (point B)), which may have resulted from the covering of molybdenum and titanium particles by ductile tantalum. Separately, fragments of Sn and Ta in particles were not observed. The results of the EDS analysis revealed that after 10 h of milling, Sn did not show a high solubility in α-Ti (Figure 5 (point D)). 

At this stage of the milling process, the collision force between the starting powders and the balls was a predominant factor in the deformation process. A progressive ball milling time led to the creation, refinements and homogenization of layered particles that were formed from different compositions of the initial components. This was due to an increase of the cold welding superiority and an increase of the coefficient of mutual diffusion of the alloying elements as a result of generating a large number of defects (i.e., dislocations and vacancies).

In general, a more homogeneous distribution is expected when the bead time increases because the homogeneity of the particles after milling is the result of a balance between cracking and cold welding processes during the MA. The increase in the milling time to above 15 h for each particle resulted in a plate-like structure consisting of a fine and relatively homogeneous distribution of solute components in the Ti matrix (Figure 5 (points E–H)).

Increasing the ball milling time to over 40 h caused significant changes in the microstructure and homogeneity of the particles because the dissolved Mo, Ta and Sn elements were deposited at interfaces and were incorporated into the Ti matrix (Figure 5 (point I–J)).

For the TTM3S powders, for milling times above 60 h, the results of the SEM-EDS studies reveal that the material was more homogenous compared to TTM powders (Figure 5 (point K–N)). It is therefore correct to state that tin, when used as a PCA, also improves the mutual solubility of the starting components.

**Figure 6:**
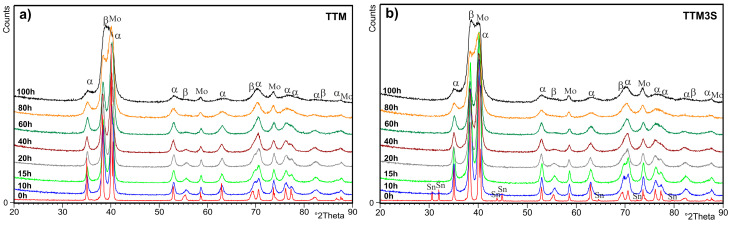
X-ray diffraction patterns and changes in the weight fractions of the α, β and Mo phases of (**a**) TTM and (**b**) TTM3S powders after 10, 15, 20, 40, 60, 80 and 100 h of milling.

In order to control the phase transformations, an analysis of the milled powders was carried out using XRD. Figure 6 show the XRD patterns of the powders after mechanical alloying according to the milling time. The X-ray phase analysis showed that the powders were free of oxides and impurities, and contained the following phases: αTi (ICDD PDF 00-044-1294), βTi (ICDD PDF 00-034-0370) and the starting element Mo (ICDD PDF 01-089-5158). As shown in Figure 6, the intensity of the XRD peaks slightly decreases with an increasing milling time. A considerable broadening of the diffraction peaks was noted because the powders were deformed as a result of the permanent collisions of balls and splitting of the powders. This can be attributed to the severity of the lattice distortions and the reduction in the crystallite size. This indicates the correct course of mechanical alloying leading to the synthesis of the β phase. Additionally, increasing the mechanical alloying time causes the shift and expansion of Ti peaks due to the accumulation of lattice defects and the supersaturation of Ta, Mo and Sn atoms.

**Figure 7:**
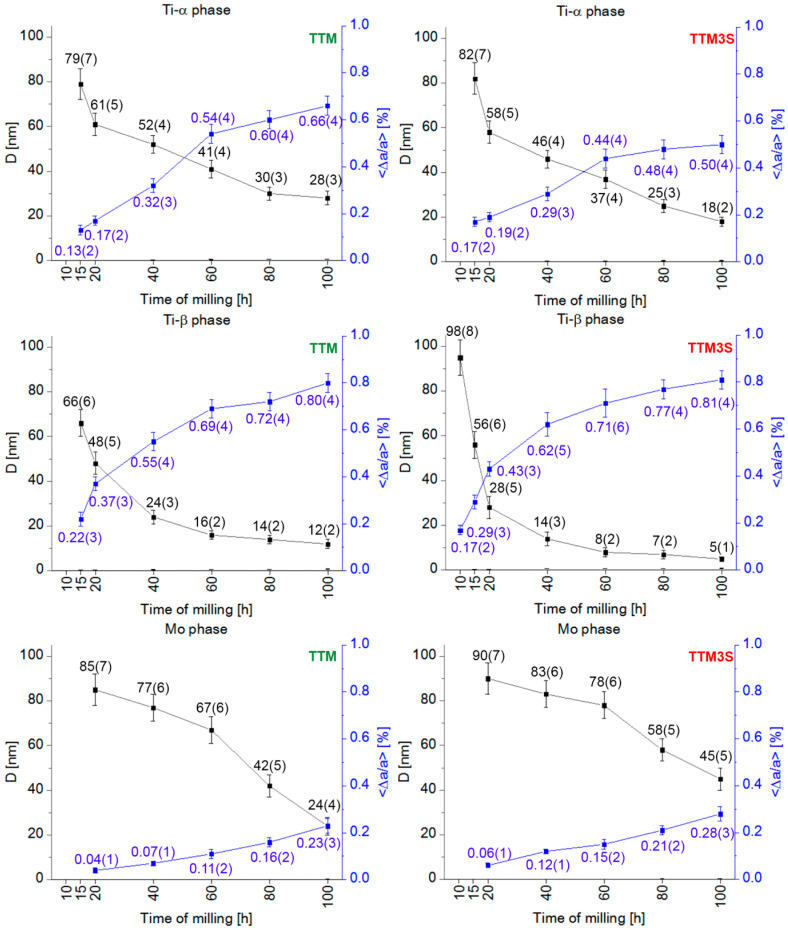
Changes in the average crystallite size (D) and lattice distortion (<Δa/a>) of the weight fractions of the α, β and Mo phases of the TTM and TTM3S powders after 10, 15, 20, 40, 60, 80 and 100 h of milling.

This statement is strongly supported by the gradually reduced crystalline-to-nanocrystalline size (Figure 7). When the milled powder is greatly deformed, not only can the grain size be reduced to nanometric dimensions, but the chemical long-range and short-range orders simultaneously decrease, leading to the formation of a nanocrystalline phase and then an amorphous phase.

Based on the Williamson–Hall method, it was concluded that after almost every milling time nanocrystalline materials were obtained. The only deviations were noted for the molybdenum phase at the two shorter milling times in both types of powders. For all phases, a reduction in the crystallite size and an increase in the lattice distortion occurred simultaneously with an increase in the milling time. Figure 7 shows that the change in the estimated crystallite size of the beta phase is logarithmic. It was found that the milling process causes a rapid reduction in the crystallite size in the early steps before stabilizing in the later steps. The average crystallite size of the β phase for the TTM powder was approximately 94(5) nm and 24(3) nm after ball milling for 10 and 40 h, respectively. The rate of the size decrease was approximately 2.3 nm/h. After subsequent ball milling from 40 to 100 h, the average crystallite size decreased from 24(3) to 12(2) nm, and the rate of size slowed down and was approximately 0.2 nm/h. Similar logarithmic trends of crystallite size variations at various milling times were observed for powders containing 3% of tin (TTM3S). An analysis of the results from Figure 7 shows that the presence of PCA increases the efficiency of the nanocrystallization process. This is particularly evident for the β phase in the TTM3S sample; it was noted that a reduction in the crystallite size took place simultaneously with the presence of tin, finally reaching 5(1) nm after the longest milling time. The average crystallite size of the β phase for the TTM3S sample was approximately 98(8) nm and 14(3) nm after ball milling for 10 and 40 h, respectively. The rate of the size decrease was approximately 2.8 nm/h. 

After subsequent ball milling from 40 to 100 h, the average crystallite size decreased from 14(3) to 5(1) nm, and the rate of the size decrease slowed down and was approximately 0.15 nm/h. After 100 h of milling, the lowest values of the crystallite sizes for the α phase were estimated at values of 28(3) nm and 18(2) nm, respectively for the TTM and TTM3S powders. For the α phase, an increase in the dispersion of the crystallites was also noticed. After 100 h of milling, the lowest values of the crystallite size for the α phase were estimated at 41(4) nm and 37(4) nm, respectively for TTM and TTM3S powders.

The continuous increase in the lattice distortion of the α and β phases can be observed, which for the β phase ultimately achieved the same value, 0.80%, for the TTM and TTM3S powders after ball milling for 100 h. The crystallite size of molybdenum after 10 h of milling was over 100 nm, and hence no data point is provided on the graph. It can be observed that milling leads to a rapid reduction in the Mo crystallite sizes. For the TTM sample, the average crystallite size of the Mo phase was approximately 85(7) nm and 24(4) nm after ball milling for 20 and 100 h, respectively. The rate of the size decrease was approximately 15 nm/h. Ball milling of the TTM3S powder to 100 h caused the rate of the size decrease to become approximately 9 nm/h, and the average crystallite size decreased from 90(7) nm to 45(5) nm for powder milled for 20 and 100 h, respectively. The lattice strains <Δa/a>, calculated from the X-ray broadening exhibited for both types of powders, were comparable and demonstrated the same upward trend. The total lattice distortions of the Mo phase were approximately 0.23(3)% and 0.28(3)% after 100 h of ball milling for the TTM and TTM3S powders, respectively. The trends whereby the crystallite size decreased and the lattice distortion increased are typical of the milled materials [15,16,17,63,64,65]. Compared with the results of similar compositions, the resulting nanocrystalline process depends on the alloying elements. Due to the high deformation rates, bands with a high dislocation density were formed at the initial milling stage. Further milling increases the average dislocation density until it reaches a critical point, when the grain breaks down into smaller particle sizes that are separated from each other by low-angular boundaries. During the subsequent milling stages, this process is repeated, except that the deformation is concentrated in areas that have not been previously deformed, as a result of which the size of the grains constantly decreases. Subsequently, grain boundaries with a small angle of inclination are replaced by grain boundaries with a larger angle of inclination, resulting in nanocrystalline particles [1,66,67].

**Figure 8:**
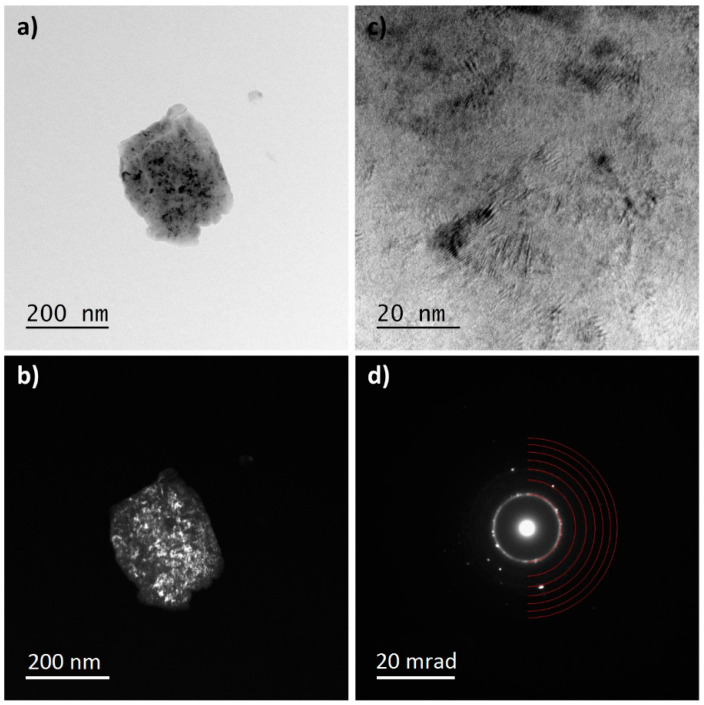
TEM analysis of the TTM powder milled for 100 h; (**a**) TEM bright-field image; (**b**) TEM dark-field images; (**c**) HR-TEM image; and (**d**) SAED pattern of the β phase.

**Figure 9:**
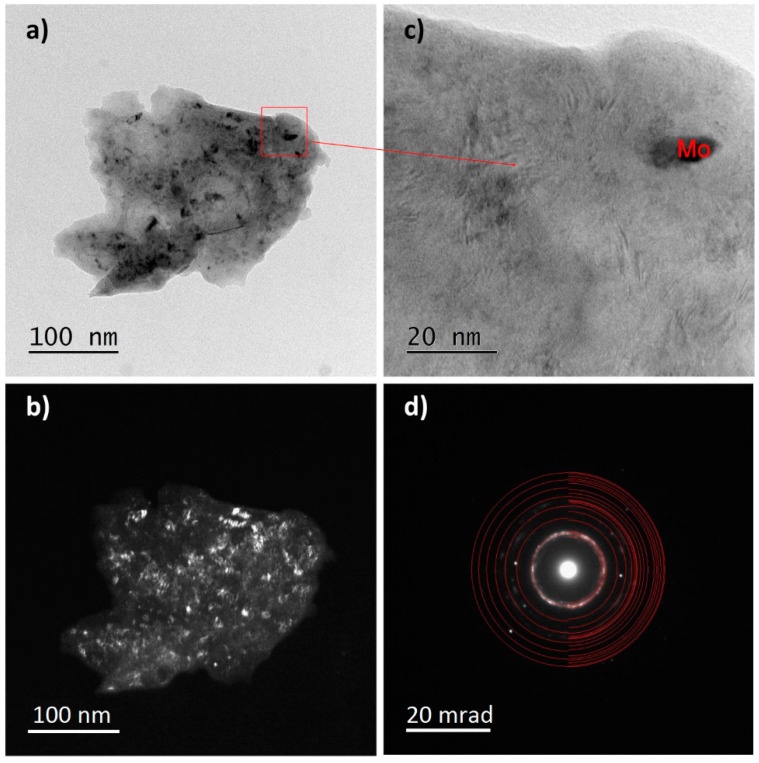
TEM analysis of the TTM powder milled for 100 h; (**a**) TEM bright-field image; (**b**) TEM dark-field images; (**c**) HR-TEM image; and (**d**) SAED pattern of the α and β phases.

**Figure 10:**
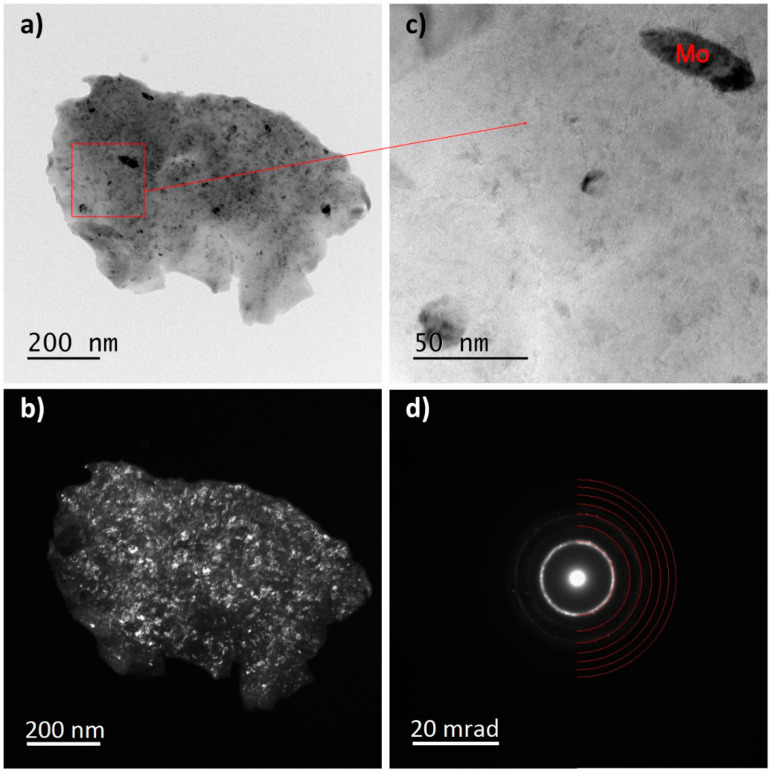
TEM analysis of the TTM3S powder that was milled for 100 h; (**a**) TEM bright field image; (**b**) TEM dark field images; (**c**) HR-TEM image; and (**d**) SAED pattern of the β phase.

The influence of the longest milling time (100 h) on the nanocrystallization of the TTM and TTM3S powders was also analyzed using the TEM method. Figure 8, Figure 9 and Figure 10 show the TEM images of the TTM and TTM3S powders after milling. Both materials presented the same type of microstructure, which was characterized by big particles.

The analysis of the recorded ring-shaped Selected-Area Electron Diffraction (SAED) patterns revealed that in both materials the α + β phases or only the β phase were present. The study of the diffraction images and bright- and dark-field images confirms the nanocrystallization of the TTM and TTM3S powders. It was found that nanocrystalline molybdenum and strongly distorted nanocrystallites were present as remaining phases. Visible Mo nanocrystallites were elongated and their grain boundaries were clearly marked for both types of powders. The lack of full alloying even during the long-term milling of molybdenum results from its lower ductility when compared to the titanium and titanium alloy. The electron microscopy results were in good accordance with the XRD studies.

**Figure 11:**
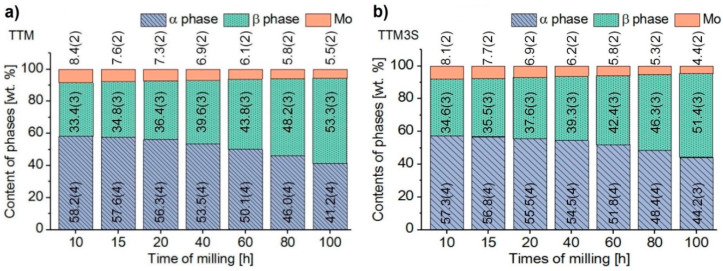
Changes in the weight fraction contents of the α, β and Mo phases of (**a**) TTM and (**b**) TTM3S powders after 10, 15, 20, 40, 60, 80 and 100 h of milling.

Rietveld refinements were used for the investigation of the milling process effect and the addition of Ta, Mo and Sn to the structure. Rietveld refinement was also used to determine the quantitative content of individual phases (Figure 11). Quantitative analysis has shown that, as the milling time increased, the content of the α and Mo phases were reduced. The analysis of the results did not show any significant effect of the PCA agent on the change in the phase contents during milling, but a slight improvement in the solubility of molybdenum for the sample containing a PCA was noted.

**Figure 12:**
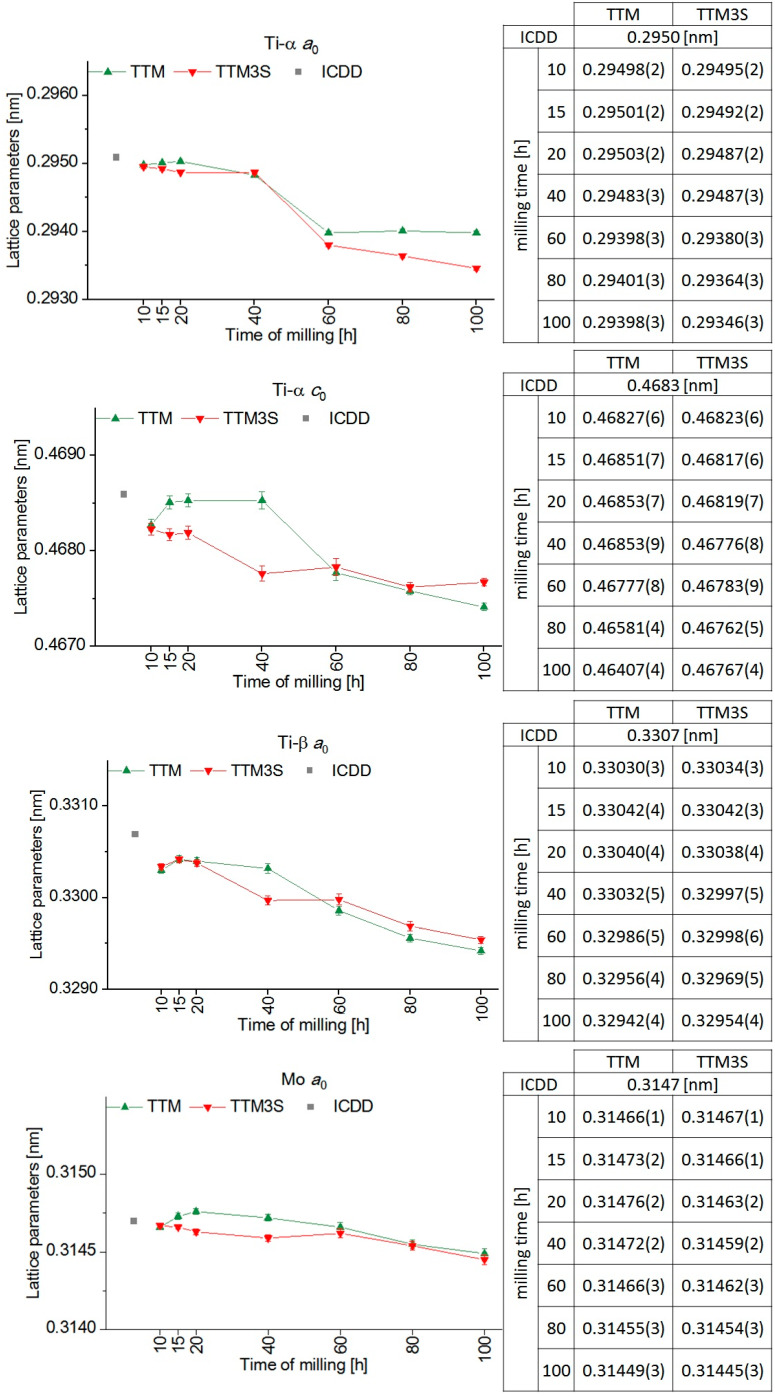
The unit-cell parameters of the α, β and Mo phases of the TTM and TTM3S powders after 10, 15, 20, 40, 60, 80 and 100 h of milling.

The changes in the lattice parameter for each phase as a function of the milling time with and without a PCA and the corresponding ICDD data sheets are shown in Figure 12. During the milling without a PCA, the lattice parameters and unit cell volume first decreased, then increased and, finally, were reduced. It should be emphasized that for the TTM3S powder containing Sn as the PCA the above changes were observed only for the β phase.

This variable for TTM and TTM3S may be the result of the appearance of a large number of defects in the ordered lattice, such as vacancies, stacking faults and dislocations [68,69]. For all the phases when the milling times were increased, a decrease of the lattice parameter was observed due to alternating cold welding and particle build-up during the milling process. A similar behavior was previously observed in other titanium alloys obtained by mechanical alloying [11,16,17,63,64,65].

In the case of the *a*_0_ parameter of the α phase, the lattice parameters of the TTM and TTM3S powders did not deviate significantly, and the deviations were within the uncertainty limits. For other lattice parameters, the β and Mo phases clearly showed that the tin caused a greater reduction in the unit cell of the individual phases. The biggest decrease of the lattice parameters was visible for a milling time of 40 h.

## 4. Conclusions

In this work, the effect of using tin as a PCA during the milling of titanium alloys was assessed. Nanocrystallization, improvement of homogenization and maximization of the process yield were considered as the primary aims. The addition of 3 wt % Sn allowed for an increased efficiency of the milling process, while maintaining a stable microstructure. Tin provides an effective method of controlling mechanical alloying without introducing the contamination of oxygen or carbon into the titanium alloy. The results showed that the samples prepared by 10 and 15 h of ball milling using Sn as a PCA had a higher yield of powder than the samples prepared without a PCA. After each milling time, greater yields were obtained for powders with Sn than for powders without Sn. A small addition of tin (3 wt %) as the PCA improved the nanocrystallization of the α and β phases of titanium (decrease of the crystallite size (D) in comparison to samples without Sn) and, simultaneously, allowed for a considerable refinement of particles and their homogenization by diminishing the cold welding and agglomeration of the pulverized particles. The X-ray diffraction and TEM studies confirmed the nanocrystallization of the new alloys Ti10Ta8Mo and Ti10Ta8Mo3Sn, as well as the transformation from the α-Ti phase to the β-Ti phase after the ball milling process. It should be mentioned that TEM and XRD tests showed the presence of nanocrystalline molybdenum particles due to their lower plasticity compared to titanium. For all the phases, increasing the milling times resulted in a decrease in the lattice parameters. In addition, the SEM-BEI results confirmed that the use of Sn as a PCA provided a better homogenization of samples prepared with at least 60 h of ball milling.

## Figures and Tables

**Table 1:** Experimental parameters of the ball milling process.

Parameters	Values
Rotation speed [rpm]	250
Milling bowl volume, [cm^3^]	80
Milling balls	Steel (AISI 52100)
Ball to powder weight ratio	10:1
Rotation speed [rpm]	200
Ball size, [mm]	10
Milling time, [h]	10; 15; 20; 40; 60; 80; 100

**Table 2:** Chemical composition of the AISI 52100 steel balls.

**Element**	Fe	Cr	C	Mn	Si	S	P
**Content (%)**	96.5–97.32	1.30–1.60	0.980–1.10	0.250–0.450	0.150–0.300	≤0.0250	≤0.0250
